# Identification of Key Genes in the HBV-Related HCC Immune Microenvironment Using Integrated Bioinformatics Analysis

**DOI:** 10.1155/2022/2797033

**Published:** 2022-10-15

**Authors:** Wei Ding, Zheng Zhang, Nianyuan Ye, Ling Zhuang, Zhiping Yuan, Wenbo Xue, Yulin Tan, Xuezhong Xu

**Affiliations:** ^1^Department of General Surgery, Wujin Hospital Affiliated to Jiangsu University, Changzhou 213017, China; ^2^Department of General Surgery, The Wujin Clinical College of Xuzhou Medical University, Changzhou 213017, China; ^3^Changzhou Key Laboratory of Molecular Diagnostics and Precision Cancer Medicine, Changzhou 213017, China; ^4^Department of Gastroenterology, Wujin Hospital Affiliated with Jiangsu University, Changzhou 213017, China

## Abstract

**Purpose:**

Hepatocellular carcinoma (HCC) has poor prognosis and high mortality among gastrointestinal tumors because of its insidious onset and strong invasiveness. However, there was little understanding of their pathogenesis. The purpose of this study was to use bioinformatics analysis to identify genes associated with the immune microenvironment in HBV-related HCC and to develop new therapeutic targets to prevent and treat cancer.

**Methods:**

RNA-seq data of HBV-related HCC cases were downloaded from TCGA-LIHC database. ESTIMATE and Deseq2 algorithms were used to screen out differentially expressed genes (DEGs). WGCNA was used to construct gene coexpression networks. In key modules, functional enrichment analysis was performed. Protein-protein interaction (PPI) was used to screen hub genes, and survival analysis was conducted to assess their prognostic significance. Following, we search for key genes differentially expressed between cancerous and paracancerous tissues in GSE136247 and GSE121248 datasets. Reveal the potential links between key genes in immune infiltration by using TIMER. Finally, in TCGA-LIHC database, integration of key genes with clinical data were used to further validate their correlation with prognosis.

**Results:**

In the cohort of HBV-related HCC patients, immune/stromal/ESTIMATE scores were not significantly associated with patient prognosis. After bioinformatics analysis, screening out five key genes was significantly related to the prognosis of HBV-related HCC. Downregulation of SLAMF1 and TRAF3IP3 suggested poor prognosis and was related to a variety of immune cell infiltration. Furthermore, compared with adjacent nontumor tissues, TRAF3IP3 and SLAMF1 were highly expressed in tumor tissues and were linked to tumor recurrences.

**Conclusion:**

In conclusion, SLAMF1 and TRAF3IP3 were identified with higher expression in tumor tissues and associated with tumor recurrence. It will be a new research direction of tumor progress and treatment.

## 1. Introduction

About 90% of the pathological types of liver cancer were hepatocellular carcinoma (HCC) in clinical. Both morbidity and mortality are far higher than other types of tumors [1]. Gender differences (predominant in males) and geographic differences (mainly East Asia) influence the incidence of HCC [2]. The main causative factors associated with HCC are virus (chronic hepatitis B and C), metabolism (diabetes and nonalcoholic fatty liver disease), toxicity (alcohol and aflatoxins), and immune system-related diseases [3]. Affected by these factors, the morbidity of HCC is rising continuously year by year. According to statistics data, more than half of HCC patients in the world are infected with hepatitis B virus (HBV). It is the main risk factor for human [4]. Mortality associated with HCC is also increasing. Recent studies have shown that there were 85% of patients with cirrhosis infected with HBV, and less than 20% of people survived more than five years [5]. Reassuringly, the incidence of HCC was significantly reduced in the middle-aged population aged 30-59, largely due to the global hepatitis B virus vaccination program [6]. Liver transplantation and surgical resection are treatment options for HCC in early-stage HCC and when the tumor size is <5 cm [7]. However, because the disease is mostly asymptomatic in its early stages, most symptomatic patients are diagnosed at an advanced stage. Currently, patients with advanced HCC are mostly treated with radiofrequency ablation (RFA), transhepatic arterial chemotherapy and embolization (TACE), tyrosine kinase inhibitor (TKI), and immunotherapy, but with the emergence of drug resistance and disease recurrence, these modalities do not significantly prolong lifespan [8]. As research progresses in depth, new and diverse avenues for the treatment of HCC are being discovered.

The current study suggests that HCC is caused by HBV-induced DNA damage that triggers hepatocyte regeneration and chronic inflammation in the liver [9, 10]. The nucleocapsid of HBV-infected hepatocytes allows the virus to replicate stealthily without being recognized by type I IFN [11]. It is now widely believed that the immune pathogenic mechanism of HCC is mainly that HBV, as a noncytopathic virus, promotes the disorder of the liver immune system and causes liver damage through abnormal immune attack. It is increasingly believed that immune pathogenesis significantly influenced the development of HBV-related HCC [12]. Although HBV was thought to contribute to HCC, there was still no clear understanding of the mechanism.

In this study, RNA-seq data and clinical feature information of HBV-related HCC patients were accessed by TCGA-LIHC. The prognosis-related DEGs and modules were screened by Sangerbox and WGCNA. In addition, Gene Ontology (GO) and Kyoto Encyclopedia of Genes and Genomes (KEGG) pathways were enriched for these DEGs and module genes and construct the PPI network to search the prognosis-related hub genes. Through the gene expression information of GSE136247 and GSE121248 in the GEO database, the possible key genes (SLAMF1 and TRAF3IP3) were finally locked. Through the TIMER database, the association between SLAMF1 and TRAF3IP3 and immune cell infiltration was analyzed. Finally, by analyzing the clinical characteristics of HBV-related HCC patients, it was confirmed that SLAMF1 and TRAF3IP3 were negatively correlated with the recurrence of patients.

## 2. Methods and Materials

### 2.1. Data Acquisition

We gathered gene expression RNA-seq and accompanying medical data of HBV-related HCC patients from TCGA-LIHC database (https://portal.gdc.cancer.gov/) [13]. HBV-infected and noninfected patients were differentiated based on the patient's past infection history. Download gene expression data from GSE136247 and GSE121248 datasets from GEO database (https://www.ncbi.nlm.nih.gov/geo/) [14]. The GSE136247 dataset contained 39 HCC tissues (25 with HBV infection) and 30 noncancerous normal tissues (19 with HBV infection) [15]. The GSE121248 dataset contained cancer and normal tissues from 37 HCC cases, and these patients had a history of HBV infection [16].

### 2.2. ESTIMATE Scores

The amount of tumor cells, immune cells, and stromal cells was determined using ESTIMATE (Estimation of STromal and Immune cells in MAlignant Tumor tissues using Expression data) based on the transcriptional profile of cancer samples. According to the stromal signature (stromal signature gene) and immune signature (immune cell signature gene), the stromal score and immune score were calculated by ssGSEA, respectively. Finally, the two scores were combined to generate an ESTIMATE score, which was used to analyze tumor purity. From the official website (https://bioinformatics.mdanderson.org/estimate/), matrix, immune, and ESTIMATE scores were downloaded for each sample in TCGA-LIHC cohort, and non-HBV-infected samples were excluded. In addition, this score was compared with tumor patient survival in a correlation analysis.

### 2.3. Acquisition of DEGs Based on Immune and Matrix Scores

All HBV-related HCC patients were divided into two groups (high vs. low) with positive and negative values. Data analysis was performed on Sangerbox [17] using the “Deseq2” package. The filter range for DEGs were determined to be log|*FC*| > 1, *P* < 0.05.

### 2.4. Gene Ontology (GO) and Kyoto Encyclopedia of Genes and Genomes (KEGG) Function Analysis

Analyze the biological functions of DEGs by using the GO enrichment analysis (including BP, CC, and MF) and KEGG pathway enrichment analysis from DAVID online website tools (database annotation, visualization, and comprehensive discovery, https://david.ncifcrf.gov/tools.jsp) [18].

### 2.5. Weighted Correlation Network Analysis (WGCNA)

WGCNA is an analytical method for analyzing gene expression patterns of multiple samples, which can cluster the similar expression gene and investigate the association between specific traits and phenotypes in modules. It will help us find relevant biomarker genes and therapeutic targets. The “WGCNA” package was used to build the DEGs coexpression network on Sangerbox to identify the modules related to prognosis.

### 2.6. Construction of PPI Network and Filtration of Hub Gene

The protein information and PPI network information of key modules were analyzed using String database (https://cn.string-db.org/) [19]. MCODE was a plugin for constructing functional modules of gene (protein) network clustering in Cytoscape 3.8.0. According to the analysis results, the hub gene can be determined [20].

### 2.7. Survival Analysis

Plot the Kaplan-Meier survival curves of these hub genes, and screen out the hub genes significantly correlated with overall survival (*P* < 0.05) by log-rank test.

### 2.8. Verification of the Expression of Hub Genes

Compare the previous hub genes with the expression data in GSE136247 and GSE121248 datasets to obtain the final key genes. We used “TIMER” in Sangerbox to analyze the correlation of key genes with 6 tumor-infiltrating immune cells in HBV-related HCC tissues.

### 2.9. Clinical Features in Patients with HBC-Related HCC

For comparison, the basic information and medical data of HBV-related HCC patients were separated into high and low groups based on the expression of key genes.

### 2.10. Statistical Analysis

**Figure 1:**
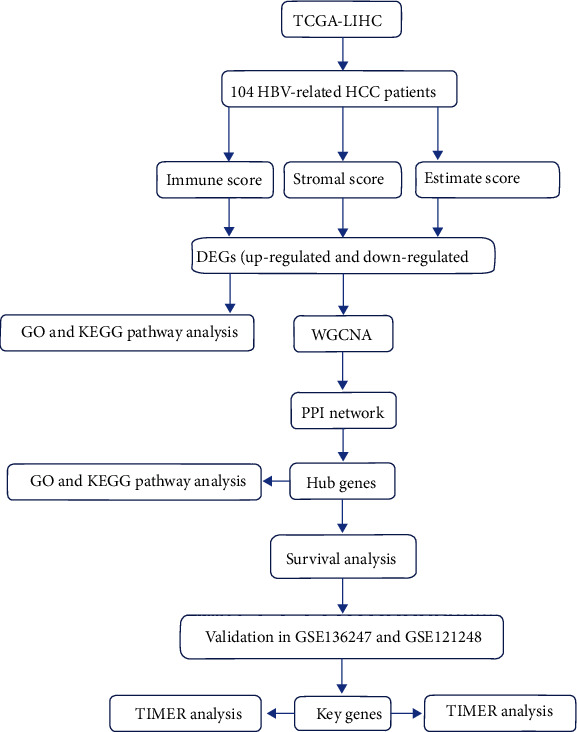
Flow chart of the study.

Analysis in the present study were conducted using the R package on Sangerbox and GraphPad prism 8.0.2. We used log-rank tests and chi-square tests for data analysis. A statistically significant difference was considered to be less than 0.05. The whole process of bioinformatics analysis was shown in Figure 1.

## 3. Results

### 3.1. Scores of the Immune System and Stroma Correlated with Overall Survival

Based on TCGA database, the statistical data of 104 HBV-related HCC patients were gained. Patients ranged from 23 to 83 years of age. 85 (81.7%) were male, and 19 (19.3%) were female. For each sample, ESTIMATE scores were calculated based on matrix, immune, and ESTIMATE scores. Stromal scores ranged from -1731.43 to 261.96, immune scores ranged from -964.97 to 2311.6, and ESTIMATE scores ranged from -2488.91 to 2306.2. In order to probe the possibility of the connection between immune/stromal/ESTIMATE scores and patient survival, we categorize HBV-related HCC patients into low and high groups on the basis of 0 scores. There were no positive results between the two groups (Figures S1A, S1B, and S1C).

### 3.2. Identification of DEGs in HBV-Related HCC

**Figure 2:**
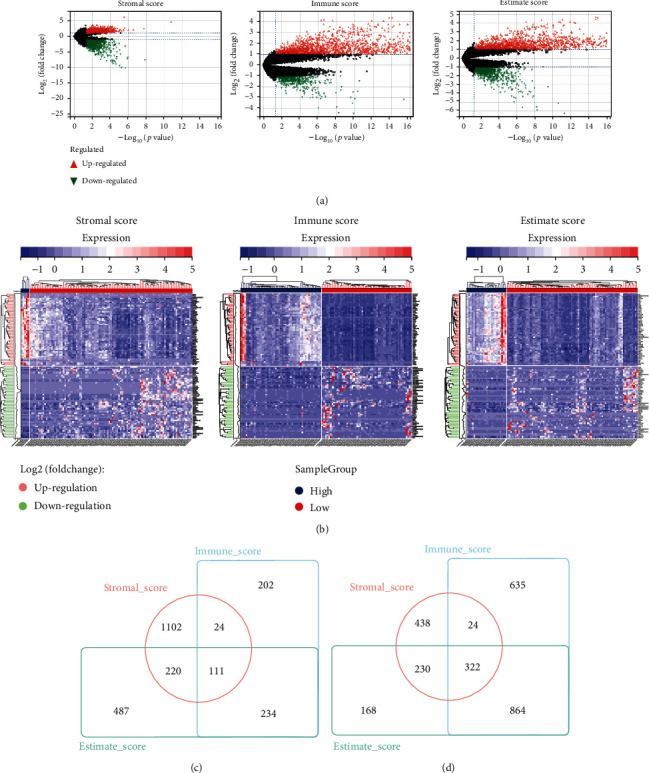
Identification of differentially expressed genes (DEGs) based on immune/stromal/ESTIMATE scores in HBV-related HCC. (a) Three respective volcano maps of the three groups. (b) Gene expression heat maps for three significantly differentially expressed groups. (c, d) Intersection of three groups of differentially expressed genes.

For expounding the connection between gene expression profiles and immune status, we used “DESeq2” package to identify. Genes were significantly differential expression among the three groups of scores. |log(*FC*)| > 1 and *P* < 0.05 were as screening criteria. As shown in Figure 2(a), 571 downregulated genes and 1,845 upregulated genes were detected in the immune score group; in the stromal score group, 1,457 downregulated genes and 1,014 upregulated genes were detected; in the ESTIMATE score group, 1,052 were detected downregulated genes and 1,584 upregulated genes. According to the heat map, there were significant differences between the three groups in the differential genes (Figure 2(b)). Through further data screening, the differential express gene in all three groups were obtained, including 111 upregulated genes and 322 downregulated genes as shown in Figures 2(c) and 2(d).

### 3.3. Functional Enrichment Analysis

DAVID website was used for GO and KEGG analyses. As a result of the enrichment analysis, cellular components (CC), molecular functions (MF), and biological processes (BP) were enriched by GO enrichment analysis (Figure S2). For BP, DEGs were mainly enriched in external encapsulating structure organization, biological adhesion, and collagen fibril organization. For CC, DEGs were mainly enriched in collagen containing extracellular matrix, external encapsulating structure, and T cell receptor complex. For MF, DEGs were mainly enriched in extracellular matrix structural constituents, glycosaminoglycan binding, and heparin binding. For KEGG, DEGs were mainly enriched in the regulation of hematopoietic cell lineage, cytokine-cytokine receptor interaction, and viral protein interaction with cytokine-cytokine receptor (Figure S2).

### 3.4. Weighted Correlation Network Analysis

**Figure 3:**
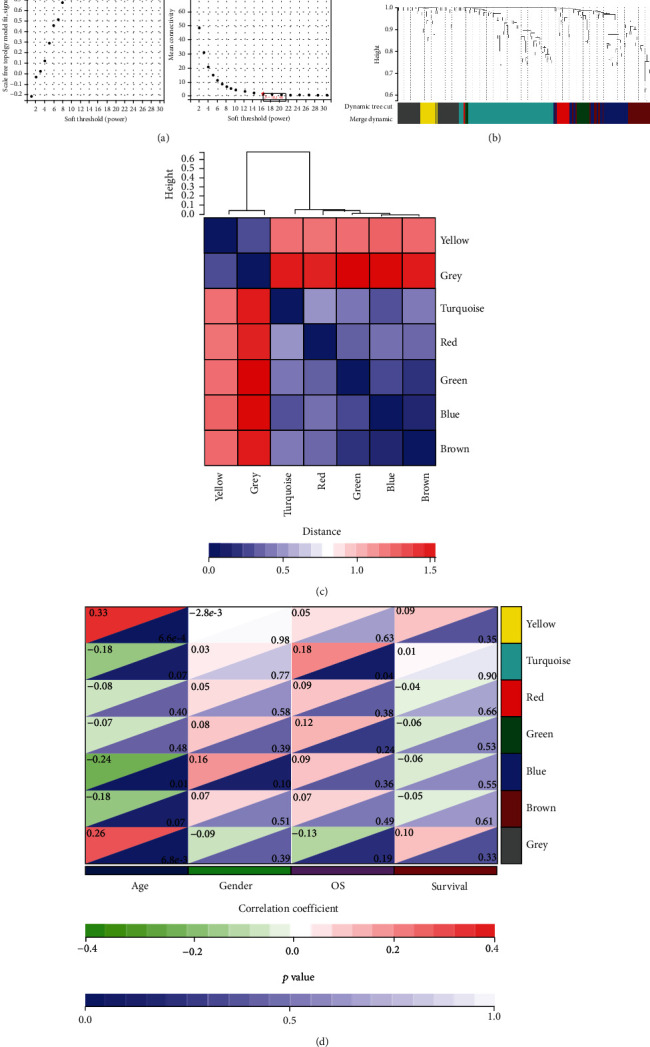
Weighted correlation network analysis (WGCNA). (a) Analysis of the scale-free fit index (left) and the mean connectivity (right) for various soft-thresholding powers. (b) Gene clustering dendrograms. (c) Topological overlap heat maps. (d) Heat map of correlations between modules and clinical features.

The role network of DEGs was constructed by WGCNA analysis. With the network's soft threshold set at 16, coexpression networks resembled scale-free networks most closely (Figures 3(a)–3(c)). According to different functions, DEGs can be divided into 7 modules. Turquoise was the module with the highest significant difference over survival (OS time) (Figure 3(d)). The module contained a total of 50 genes.

### 3.5. Functional Enrichment Analysis of Genes in Turquoise

**Figure 4:**
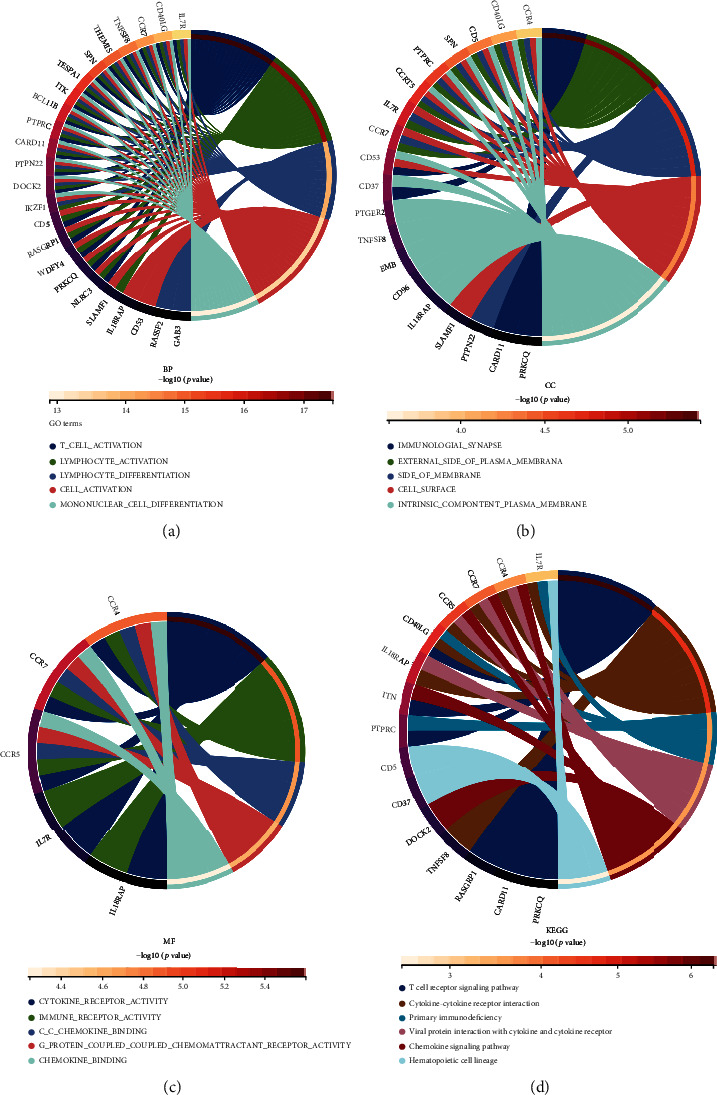
Chord diagram demonstrates GO and KEGG analysis of module genes. (a) Biological processes (BP), (b) cellular components (CC), (c) molecular functions (MF), and (d) KEGG pathways.

These genes were mostly associated with T cell activation, lymphocyte activation, and leukocyte differentiation in BP, based on GO enrichment analysis (Figure 4(a)). For CC, these genes were mainly enriched in immunological synapse, external side of plasma membrane, and side of membrane (Figure 4(b)). For MF, these genes were mainly enriched in cytokine receptor activity, immune receptor activity, and C-C chemokine binding (Figure 4(c)). For KEGG, these genes were mainly involved in the regulation of T cell receptor signaling pathway, cytokine-cytokine receptor interaction, and primary immunodeficiency (Figure 4(d)).

### 3.6. Filtration of PPI Network and Identification of Prognostic-Related Genes

**Figure 5:**
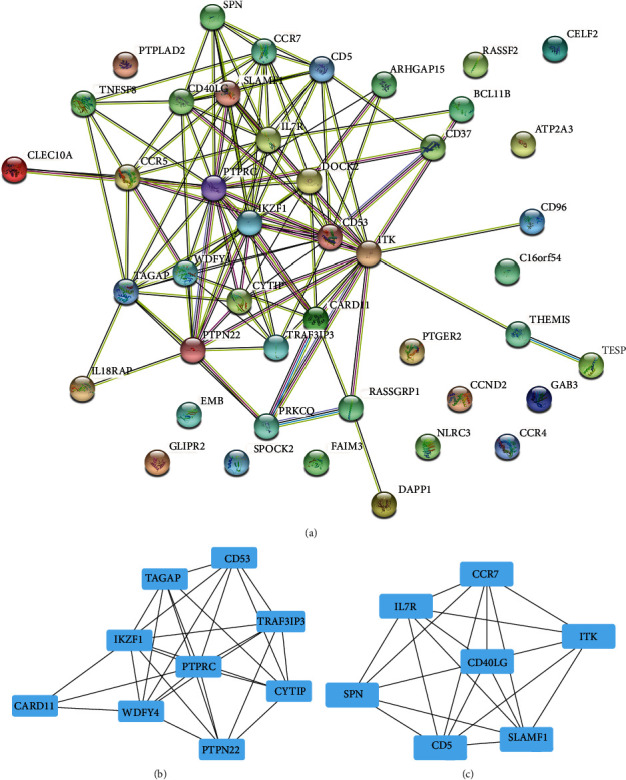
Construction of PPI network and identification of key subnetwork. (a) PPI network was constructed based on the STRING database and Cytoscape software. (b) The subnetwork contains 9 nodes and 31 edges. (c) The subnetwork contains 7 nodes and 23 edges.

Through the String database, a PPI network was constructed using 50 genes (Figure 5(a)). Then, use Cytoscape 3.8.0 to further optimize the obtained PPI network, and use the MCODE plugin to draw important subnetworks (Figures 5(b) and 5(c)). There were 16 central genes (CD53, TAGAP, IKZF1, CARD11, WDFY4, PTPRC, PTPN22, CYTIP, TRAF3IP3, CCR7, ITK, IL7R, CD40LG, SLAMF1, CD5, and SPN) in the protein interaction network.

### 3.7. Survival Analysis in Blue Module

**Figure 6:**
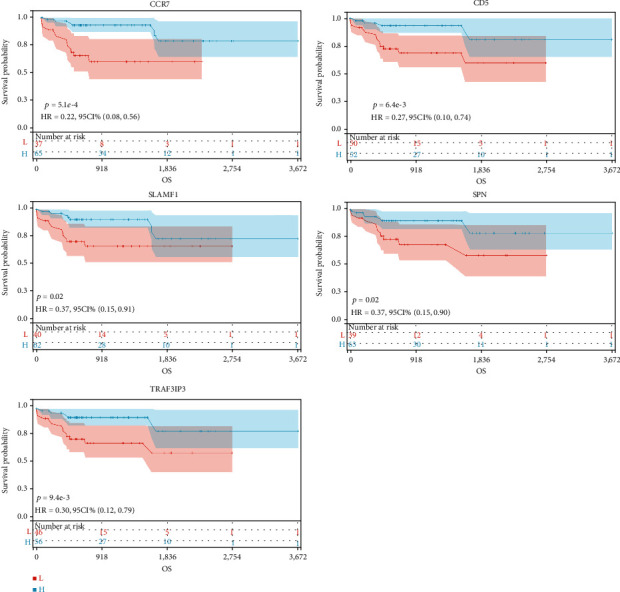
Survival analysis. The relations between the expression levels of CCR7, CD5, SLAMF1, SPN, and TRAF3IP3 and OS in HBV-related HCC.

Identifying genes associated with overall survival in patients with HBV-related HCC was the purpose of this research. We constructed Kaplan-Meier survival curves of these genes using the prognostic information in TCGA-LIHC. Among them, the expression levels of CCR7, CD5, SLAMF1, SPN, and TRAF3IP3 were significantly associated with the prognosis of patients (Figure 6 and Figure S3).

### 3.8. Validation of the Analysis in the GEO Database

**Figure 7:**
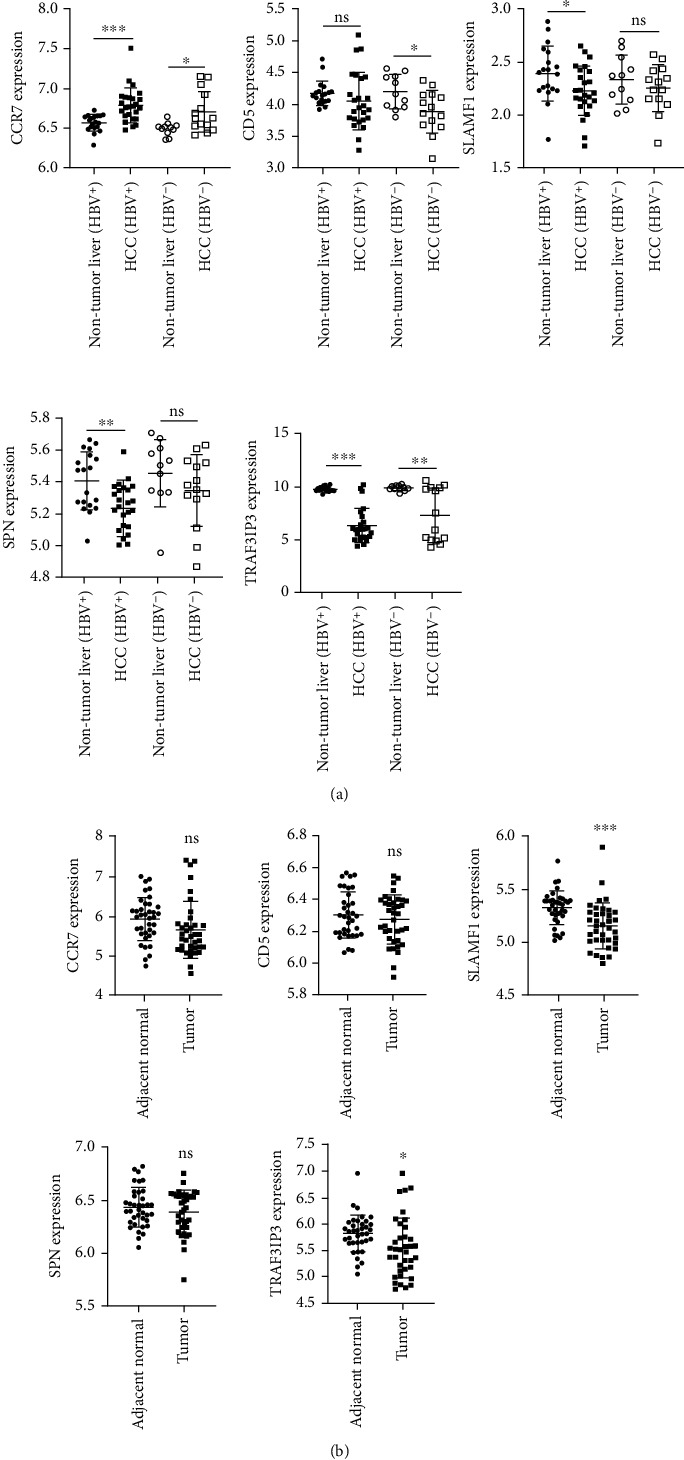
Hub genes were validated in the GEO database. (a) The expression levels of CCR7, CD5, SLAMF1, and SPN in GSE136247. (b) The expression levels of CCR7, CD5, SLAMF1, and SPN in GSE121248. ^∗^*P* < 0.05, ^∗∗^*P* < 0.01, ^∗∗∗^*P* < 0.001; ns: not significantly.

In addition, we determined the use of GSE136247 and GSE121248 to explore the expression of these genes in cancerous and paracancerous tissues. As shown in Figure 7(a), in GSE136247, CCR7 was expressed significantly upregulated in cancer tissues (whether or not infected with HBV) relative to adjacent tissues, whereas TRAF3IP3 was completely the opposite. In HBV-related HCC patients, SLAMF1 and SPN in cancer tissues were significantly decreased, but this phenomenon was not observed in patients without HBV infection. In addition, the expression of CD5 was significantly decreased in cancer tissues relative to adjacent tissues of HBV-uninfected patients, while in HBV-related HCC patients, the two groups did not differ significantly. As shown in Figure 7(b), in GSE121248, the expressions of SLAMF1 and TRAF3IP3 were significantly decreased in HBV-related HCC tissues relative to paracancerous tissues, while the expressions of CCR7, CD5, and SPN were not significantly different. Based on the above results, we defined SLAMF1 and TRAF3IP3 as key genes for follow-up studies.

### 3.9. Connection between Key Genes and Immune Infiltration

**Figure 8:**
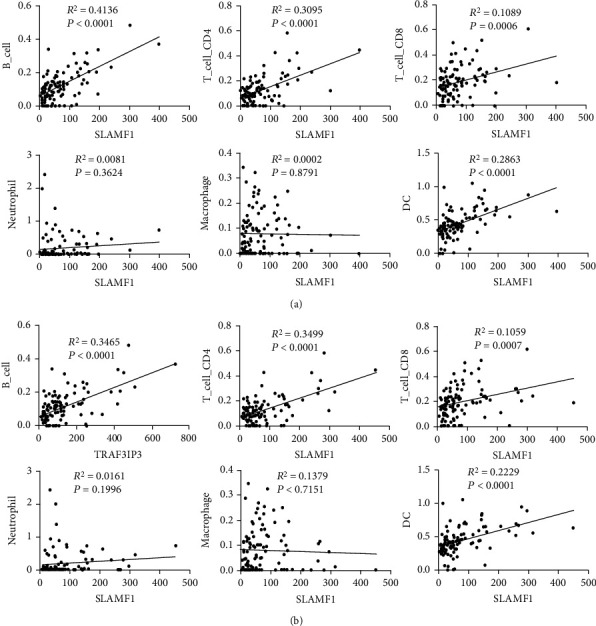
The correlation between key genes and 6 immune cell types. (a) SLAMF1; (b) TRAF3IP3.

In the present study, we explored possible associations between key gene expression and the infiltration of immune cells using TIMER. A positive correlation was found between SLAMF1 and TRAF3IP3, but not between neutrophils and macrophages, with the infiltration of B cells, CD4+ T cells, CD8+ T cells, and dendritic cells (Figure 8). In light of this, key genes may play an important role in regulating immune cells.

### 3.10. Relationship between Key Genes and Clinical Features

Based on TCGA-LIHC database, we examined the relationship between SLAMF1 and TRAF3IP3 and HBV-related HCC clinical characteristics. The results showed that SLAMF1 and TRAF3IP3 were inversely associated with tumor recurrence, regardless of gender, age, tumor stage (T), lymph node stage (N), and metastasis stage (M) (Table 1).

## 4. Discussion

HCC has a poor prognosis, killing more than 800,000 people worldwide each year [21]. In European and American countries, the prevalence of nonalcoholic fatty liver disease is rising rapidly every year, leading to the subsequent development of HCC and HCC-related death; while in developing countries in Asia, hepatitis and cirrhosis caused by viral infection are the main causes of HCC [22]. As most HCC cases are secondary to hepatitis (hepatitis B, hepatitis C, or alcoholic and nonalcoholic liver disease) or cirrhosis, HCC is now gradually considered to be the inflammatory cancer induced by chronic liver injury [23–26]. Patients with advanced HCC lack access to surgery and rely mainly on immunization or chemotherapy, example for sorafenib, a kind of the tyrosine kinase inhibitor. In recent years, several treatment options (lenvatinib, regorafenib, cabozantinib, and ramucirumab) have emerged for the various aspects' treatment of advanced HCC [27].

Cellular components of the HCC immune microenvironment (tumor cells, immune cells, stromal cells, endothelial cells, and cancer-associated fibroblasts) are critical for the response to immunotherapy [28]. Through the portal vein, antigen-rich blood from the gut is constantly exposed to the liver, which acts as a central immune organ. In order to reduce inflammatory stimulation and tissue damage from the blood and the liver, establish an immune-tolerant microenvironment which has a strong resistance to hit and self-cleaning ability. The homeostasis of this immune microenvironment is also disrupted when hepatitis or cirrhosis or even HCC develops [29]. The TME in HCC is the hallmark of tumor, which has an important influence on tumor growth, invasion, and drug resistance [30, 31].

First, we screened out HCC patients with HBV infection from TCGA-LIHC. In the immune microenvironment of HBV-related HCC, DEGs were identified based on immunity, stroma, and ESTIMATE scores. Activation of CD4+ T cells, CD8+ T cells, NK cells, NKT cells, monocytes/macrophages, and HSCs occurs in chronic hepatitis caused by HBV. Hepatitis is further aggravated, and HCC is further encouraged by the simultaneous production of TNF-*α*, IFN-*γ*, IL-12, IL-4, and IL-13 [32, 33]. Additionally, several immunosuppressive cells, including Treg, Breg, MDSC, and Kupffer cells, inhibit immune cell activity by producing cytokines such as TGF-*β* and IL-10 and inducing key factors in CD8+ T and NK cell depletion, leading to immune escape of HBV and HCC tumor cell [34–37].

Additionally, we analyzed DEG enrichment. These DEGs have various biological properties and participate in various signaling pathways, such as external encapsulating structure organization, biological adhesion, T cell receptor complex, cytokine-cytokine receptor interaction, and viral protein interaction with cytokine-cytokine receptor. All of these confirmed that their involvement was in the regulation of the immune microenvironment in HBV-related HCC [38, 39]. Coexpression networks were constructed, with turquoise modules identified as key modules by WGCNA. Their main functions are to activate T cells, activate lymphocytes, and differentiate leukocytes; they are mainly located at the immune synapse, the outer and membrane sides of the plasma membrane; they mainly regulate cytokine receptor activity, immune receptor activity, and C-C chemokine binding. They are also involved in the regulation of T cell receptor signaling pathway cytokine-cytokine receptor interactions and primary immunodeficiency. A close correlation can be found between the immune regulation of HBV-related HCC and the genes of this module.

Through the PPI construction and prognostic-related genes analysis of this module, we identified five HBV-related hub genes for the prognosis of HCC patients, namely, CCR7, CD5, SLAMF1, SPN, and TRAF3IP3. On the basis of GSE136247 and GSE121248, the expression of each gene in cancer tissue and normal tissue was verified, and two key genes were finally obtained, namely, SLAMF1 and TRAF3IP3.

The SLAMF1/CD150 receptor is a member of the cell surface receptor signaling lymphocyte activation molecule (SLAM) family and is considered a marker of activated T cells, B cells, monocytes, and DCs [40, 41]. SLAMF1 is actively involved in the regulation of different types of immune responses as well as keeping the tissue microenvironment [42]. Recent studies have demonstrated that the expression level of SLAMF1 is significantly increased in liver tissue of NASH compared with non-NASH controls and that the level of SLAMF1 was dramatically related to the seriousness of the NASH phenotype. This study was the first to identify the role of SLAMF1 in the mediating of hepatocyte death in NASH and as a measure of NASH in humans [43]. In another study, the concentration of SLAMF1 has a profound effect on the formation of cirrhosis in the plasma. But no significant difference was found between HCC and cirrhosis [44]. TRAF3IP3 (TRAF3-interacting protein 3) was identified as a TRAF3-interacting protein in original [45]. Recent studies have shown that TRAF3IP3 is involved in B and T cell development and for maintaining the functional stability of regulatory T cells [46, 47]. TRAF3IP3 has been shown to function as an oncogene in melanoma and glioma [48, 49].

Our study showed that SLAMF1 and TRAF3IP3 were lowly expressed in HBV-related HCC and positively related with the infiltration of B cells, CD4+ T cells, CD8+ T cells, and dendritic cells, but not neutrophils and macrophages. Taken together, SLAMF1 and TRAF3IP3 may contribute to the pathogenesis of HBV-related HCC. Through their effect on the immune-suppressive microenvironment, furthermore, we found that SLAMF1 and TRAF3IP3 were also associated with the recurrence of HBV-related HCC.

## 5. Conclusion

We used bioinformatics to comprehensively analyze the expression of immune microenvironment-related genes in HBV-associated HCC patients in TCGA. Further study of the screened DEGs yielded two genes related with prognosis. We explained that SLAMF1 and TRAF3IP3 were low-expressed in HBV-associated HCC tissues and were correlated with tumor recurrence. Our findings had clear implications for SLAMF1 and TRAF3IP3 as biomarkers for predicting the prognosis of HBV-related HCC patients and provide new research directions and diagnosis and treatment options for HBV-related HCC. However, follow-up clinical studies are required to confirm these opinions.

## Figures and Tables

**Table 1:** SLAMF1 and TRAF3IP3 expression and clinicopathological features in HBV-related HCC.

Variables	*n*	SLAMF1 expression		*P* value	TRAF3IP3 expression		*P* value
		Low	High		Low	High	
Gender							
Male	85	42	43	0.636	42	43	0.636
Female	19	10	9		10	9	
Age							
>60	33	20	13	0.140	20	13	0.140
≤60	71	32	39		32	39	
T							
T1-T2	91	44	47	0.374	44	47	0.374
T3-T4	13	8	5		8	5	
N							
N0	94	49	45	0.318	50	44	0.096
Nx	10	3	7		2	8	
M							
M0	89	45	44	0.780	46	43	0.402
Mx	15	7	8		6	9	
Recurrence							
Yes	44	27	17	0.047^∗^	28	16	0.017^∗^
No	60	25	35		24	36	

^∗^ represents *P* < 0.05; T: tumor stage; N: lymph node stage; M: metastasis stage.

## Data Availability

The datasets used and/or analyzed during the current study are available from the corresponding author on reasonable request.

## Supplementary Materials

Supplementary Materials Figure S1: the relationship between immune status and overall survival in HBV-related HCC. (A) Kaplan-Meier curve shows the overall survival of the high and low immune score groups. (B) Kaplan-Meier curve shows the overall survival of the high and low stromal score groups. (C) Kaplan-Meier curve shows the overall survival of the high and low ESTIMATE score groups. Figure S2: chord diagram demonstrates GO and KEGG analysis of DEGs. Biological processes (BP), cellular components (CC), molecular functions (MF), and KEGG pathways. Figure S3: survival analysis. The relations between the expression levels of CD53, TAGAP, IKZF1, CARD11, WDFY4, PTPRC, PTPN22, CYTIP, ITK, IL7R, and CD40LG and OS in HBV-related HCC.
