# Supplementary material for: Identification of Key Genes in the HBV-Related HCC Immune Microenvironment Using Integrated Bioinformatics Analysis
Source: J Oncol. 2022 Oct 15;2022:2797033. doi: 10.1155/2022/2797033 (PMC9587913; doi:10.1155/2022/2797033)
Supplement: Supplementary Materials — Figure S1: the relationship between immune status and overall survival in HBV-related HCC. (A) Kaplan-Meier curve shows the overall survival of the high and low immune score groups. (B) Kaplan-Meier curve shows the overall survival of the high and low stromal score groups. (C) Kaplan-Meier curve shows the overall survival of the high and low ESTIMATE score groups. Figure S2: chord diagram demonstrates GO and KEGG analysis of DEGs. Biological processes (BP), cellular components (CC), molecular functions (MF), and KEGG pathways. Figure S3: survival analysis. The relations between the expression levels of CD53, TAGAP, IKZF1, CARD11, WDFY4, PTPRC, PTPN22, CYTIP, ITK, IL7R, and CD40LG and OS in HBV-related HCC. [file 2797033.f1.docx]

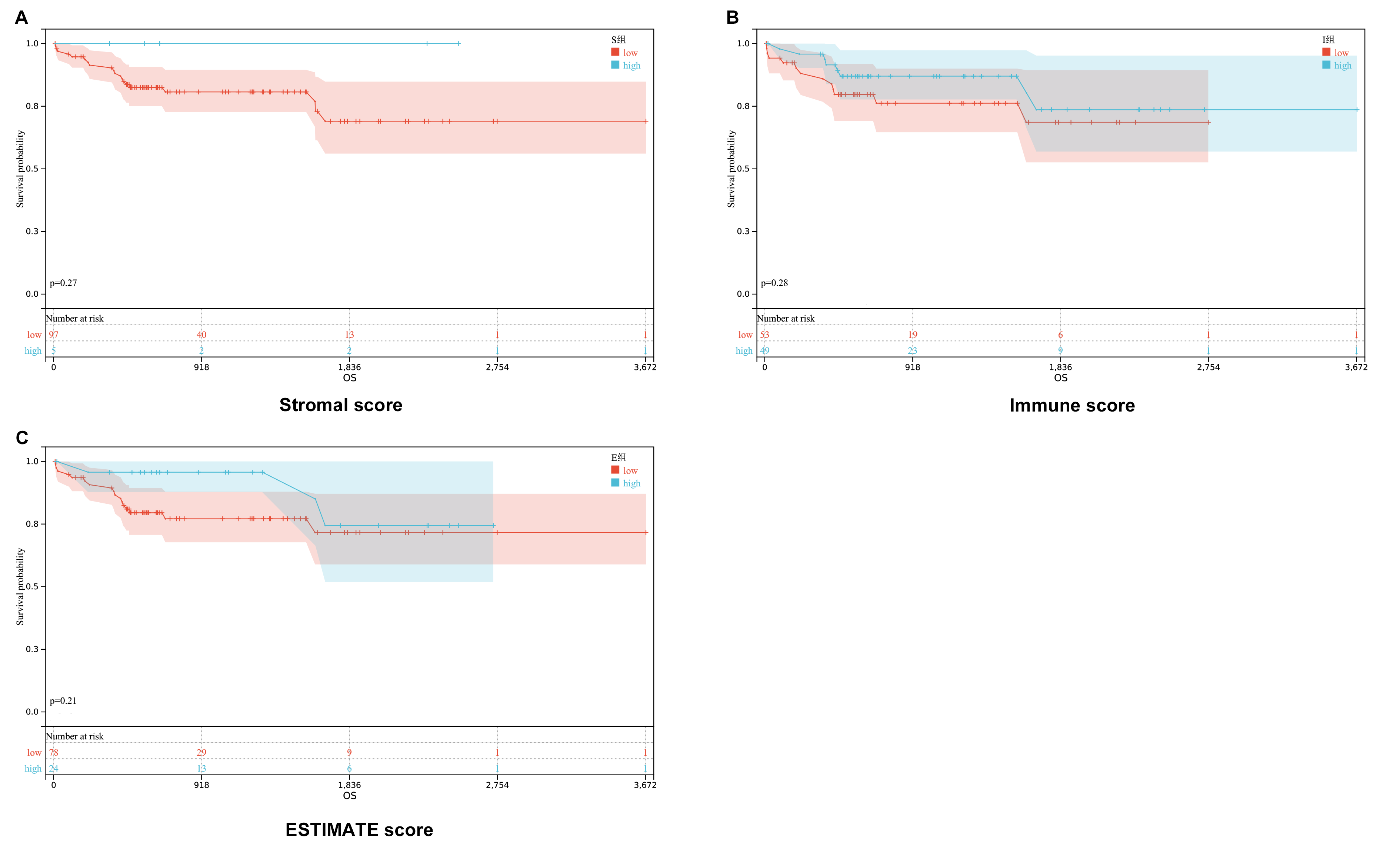


**Fig. S1 The relationship between immune status and overall survival in HBV-related HCC. (A**) Kaplan-Meier curve shows the overall survival of the high and low immune score groups. (**B**) Kaplan-Meier curve shows the overall survival of the high and low stromal score groups. (**C**) Kaplan-Meier curve shows the overall survival of the high and low ESTIMATE score groups.


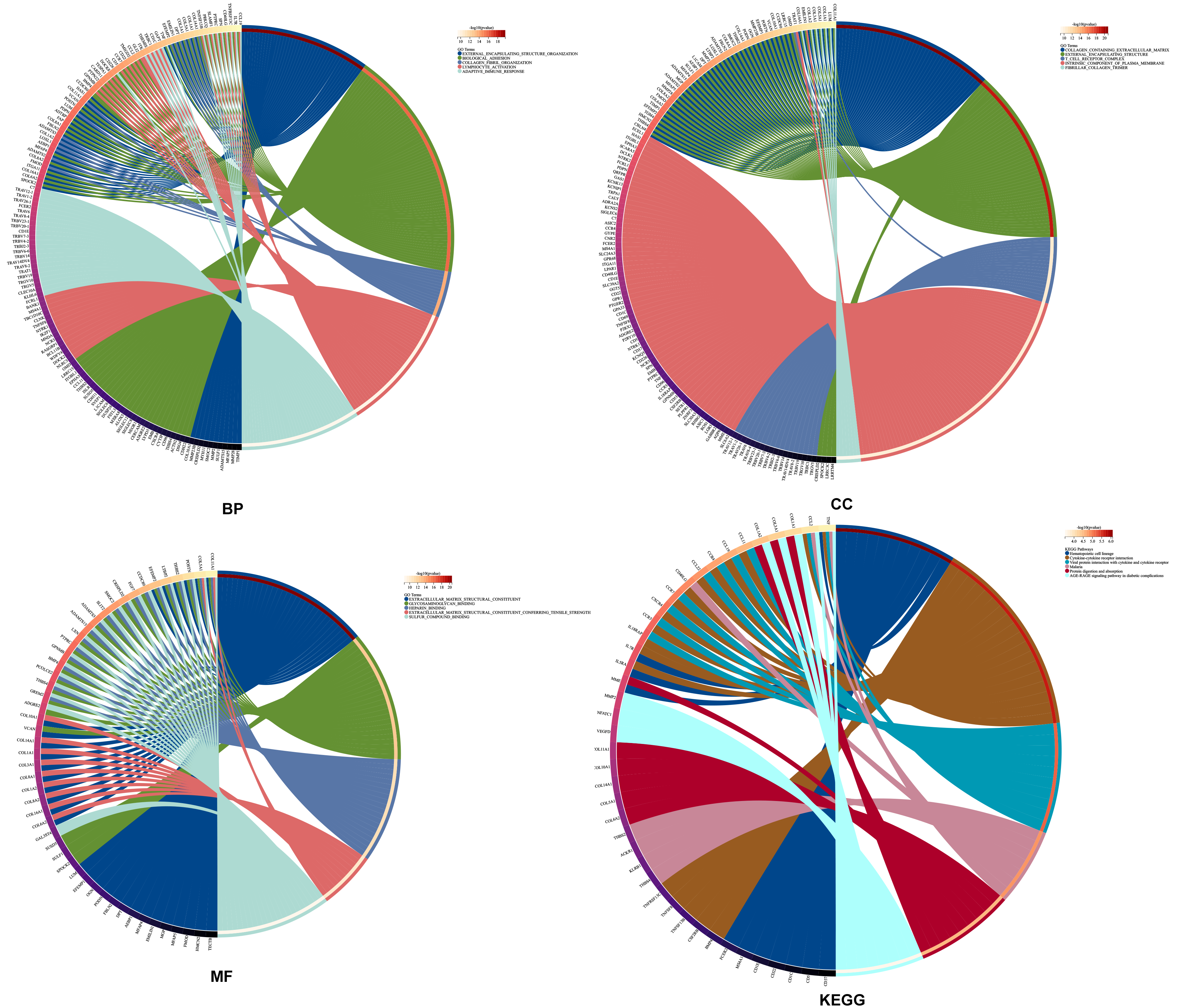


**Fig. S2 Chord diagram demonstrates GO and KEGG analysis of DEGs.** Biological processes (BP), cellular components (CC), molecular functions (MF) and KEGG pathways.


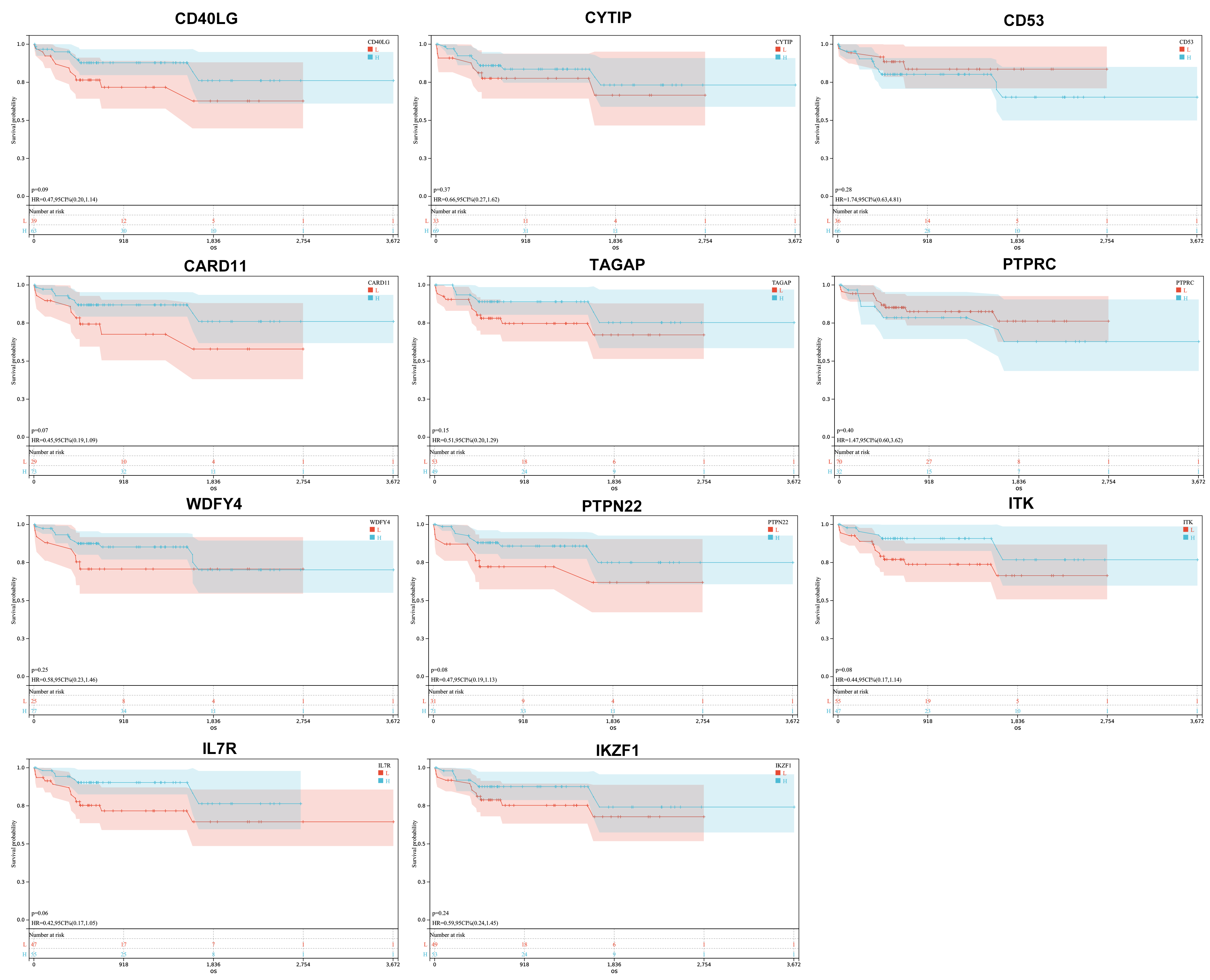


**Fig. S3 Survival analysis.** The relations between the expression levels of CD53, TAGAP, IKZF1, CARD11, WDFY4, PTPRC, PTPN22, CYTIP, ITK, IL7R and CD40LG and OS in HBV-related HCC.
